# Traumatic fracture-dislocation of the hip following rugby tackle: a case report

**DOI:** 10.1186/1758-2555-1-28

**Published:** 2009-12-15

**Authors:** Santosh Venkatachalam, Nima Heidari, Tony Greer

**Affiliations:** 1 Southend University Foundation NHS Hospital, Department of Orthopaedics, Westcliff on Sea, Essex SS0 0RY, UK

## Abstract

Posterior fracture-dislocation of hip is uncommonly encountered in rugby injuries. We report such a case in an adult while playing rugby. The treating orthopaedician can be caught unaware and injuries in such sports can be potentially misdiagnosed as hip sprains. Immediate reduction of the dislocation was performed in theatres. The fracture was fixed with two lag screws and a neutralization plate. This led to early rehabilitation and speedy recovery with return to sporting activities by 12 months.

## Background

Posterior fracture dislocation of the hip is commonly seen following high energy motor vehicle accidents. It is uncommon in sports although hip dislocations have been reported following skiing, gymnastics and even jogging. This case report highlights the magnitude of forces that can be encountered by the hip joint in a sport like rugby. Open reduction, anatomical internal fixation with good rehabilitation form the cornerstone for good recovery.

## Case report

**Figure 1:**
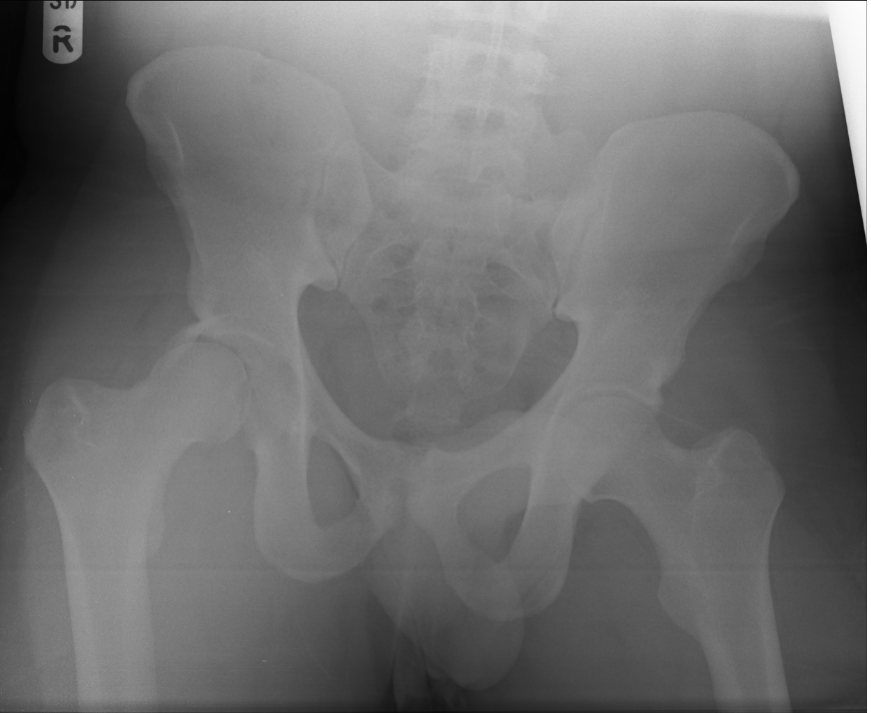
**Anteroposterior pelvis radiographs**.

**Figure 2:**
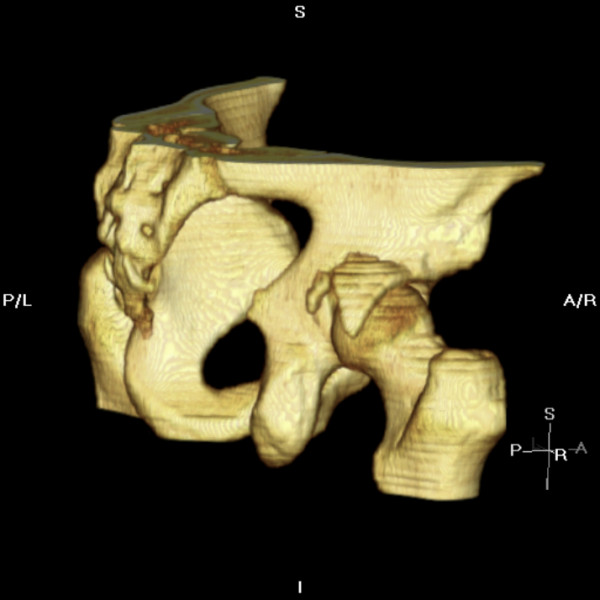
**CT scan with 3 D reconstruction**.

**Figure 3:**
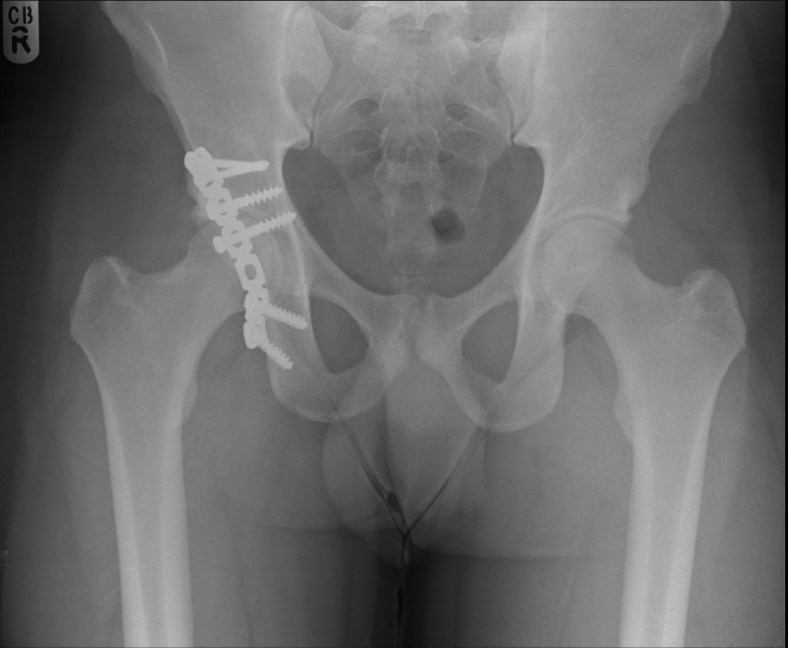
**One year follow up radiograph**.

A 28 year old amateur rugby player presented to accident and emergency with pain in the right hip following a rugby tackle. He was playing as a forward in the field and was involved in a scrum during his rugby match. He fell on to the ground when the opponent players fell over him from the front. His knee was in a flexed and adducted position with the opponent's weight falling over the front of the knee causing a posteriorly directed force on the ipsilateral hip. He heard a snap at the back of his hip and was unable to mobilize thereafter. On clinical examination, he had a tender, bruised hip with no obvious deformity to the leg. All movements of the affected hip were restricted by pain. Sciatic nerve with its common peroneal nerve component was intact clinically. Distal vascularity was well preserved. Plain radiographs of the pelvis (Fig 1) and Judet views revealed a posterior-fracture dislocation of hip. Immediate closed reduction was performed in theatres under anaesthesia within four hours. CT scan confirmed that the fragment was from the posterior wall of the acetabulum (Fig 2) and also revealed an area of indentation in the head of the femur with no incarcerated fragments in the joint. Open reduction and internal fixation of the fragment was achieved using a posterior Kocher-Langenback approach. The external rotators and the tissues were found to be edematous but intact during the operation. The area of indentation of the femoral head was small, located anteriorly and was not in the weight bearing portion. The acetabular fragment was fixed with two lag screws and a reconstruction plate in the neutralization mode, extending into the ischial tuberosity posteriorly. Post operatively, there were no sciatic nerve deficits and he was prescribed indomethacin 25 mg thrice a day for six weeks to reduce the risk of heterotrophic ossification with monitoring of his renal functions. He was mobilized non weight bearing for four weeks, allowed touch weight bearing for further four weeks and normally after that period. He was able to perform activities of daily living without any aid by three months. He returned to his occupation as a physical education teacher by six months and was playing rugby by one year. The radiographs were satisfactory at the one year follow up maintaining joint congruity with no evidence of avascular necrosis or heterotrophic ossification (Fig 3).

## Discussion

Rugby is a very popular contact sport in the United Kingdom and Europe. Head and neck are the common regions injured and these injuries are usually ligamentous in origin. Concussion in head injuries and lower limb fractures account for two percent each in this sport [1,2], and [3].

Traumatic posterior fracture dislocation of the hip is a devastating injury. The incidence of such injuries in sports is significantly lower. There are around 14 cases of traumatic hip dislocations reported in literature during recreational sports like soccer, skiing, basketball, gymnastics, equestrian sports and even jogging [4,5]. Only four have had associated fractures with the dislocation. The symptoms and signs of dislocation can be potentially misdiagnosed as a simple hip sprain.

In sports, hip subluxation is more common than dislocation indicating the lesser energy involved compared to motor vehicle accidents [6]. The common mechanism in motor vehicle accidents is due to knee hitting the dashboard with the leg in adducted and flexed position leading to a posteriorly directed force on the hip [7]. Letournel and Judet [8] have shown that the posterior acetabulum rim bears the impact of the femoral head with this leg position. The characteristic fractured acetabular posterior lip confirms the force vector and pathomechanism of injury. The indentation of the femoral head also reaffirms this mechanism. With traumatic hip dislocation, prompt reduction is of paramount importance in order to decrease the risk of subsequent osteonecrosis [9,10] as reduction may relieve tension across the femoral and circumflex vessels [11]. Also the incidence of sciatic nerve injury, post traumatic arthrosis and heterotrophic ossification comes down with emergent reduction. The radiographic workup of a patient who has a suspected hip dislocation should include radiographs of the hip, including Judet views to evaluate for posterior acetabular lip fracture.

Operative treatment is required to achieve anatomical reduction of acetabular fracture, rigid fixation and early mobilization of the joint following immediate closed reduction. In one of the studies, more than 80% had unsatisfactory results following non operative management [5]. Indications for operative management include articular displacement of > 2 mm, intraarticular fracture fragments, unstable hip after closed reduction, irreducible hip, vascular injuries, and ipsilateral femoral fractures [12]. Posterior wall acetabular fractures carry a poorer prognosis due to frequent association with direct cartilage injury, comminution, impaction and soft tissue trauma [13]. Sciatic nerve injury on presentation has been reported as high as 23% [4]. Deep vein thrombosis and deep infection are other well known complications associated with acetabular fracture surgery [14]. Indomethacin is regarded as effective prophylaxis with the reduction in the incidence of heterotrophic ossification after operation by 30-45% [15].

This case highlights the importance of prompt recognition of this devastating injury and the huge magnitude of forces across hip joint in rugby. The probable reasons for better operative results following sports injuries compared to motor vehicle accidents could be due to lesser energy involved, intact soft tissue, lower incidence of sciatic nerve damage and ability to reduce the fracture anatomically due to absence of comminution/cartilage damage. Immediate reduction of dislocation, anatomical reduction of fracture fragment, rigid fixation and early mobilization lead to good results.

## Competing interests

The authors declare that they have no competing interests.

## Consent

Written informed consent was obtained from the patient for publication of this case report and accompanying images. A copy of the written consent is available for review by the Editor-in-Chief of this journal

## Authors' contributions

SV co wrote the paper, was involved in case notes analysis and literature review, NH co wrote the paper, TG had the idea of the case report and reviewed the report critically for important intellectual content. All the authors have read and approved the final manuscript.
